# Alterations in Inflammatory Cytokines and Redox Homeostasis in LPS-Induced Pancreatic Beta-Cell Toxicity and Mitochondrial Stress: Protection by Azadirachtin

**DOI:** 10.3389/fcell.2022.867608

**Published:** 2022-06-20

**Authors:** Annie John, Haider Raza

**Affiliations:** Department of Biochemistry and Molecular Biology, College of Medicine and Health Sciences, United Arab Emirates University, Al Ain, United Arab Emirates

**Keywords:** pancreatic cell, LPS, azadirachtin, mitochondria, inflammation signaling, GSH redox metabolism

## Abstract

Inflammation and redox imbalance are hallmarks of cancer, diabetes, and other degenerative disorders. Pathophysiological response to these disorders leads to oxidative stress and mitochondrial dysfunction by alterations and reprogramming in cellular signaling and metabolism. Pancreatic beta cells are very sensitive to the inflammatory and altered nutrient signals and hence play a crucial role in diabetes and cancer. In this study, we treated insulin-secreting pancreatic beta cells, Rin-5F, with the bacterial endotoxin, LPS (1 μg/ml) to induce an inflammatory response *in vitro* and then treated the cells with a known anti-inflammatory, anticancer and antioxidant phytochemical, azadirachtin (AZD, 25 µM for 24 h). Our results demonstrated lipid peroxidation and nitric oxide production causing increased nitro/oxidative stress and alterations in the activities of anti-oxidant enzymes, superoxide dismutase and catalase after LPS treatment. Pro-inflammatory responses caused by translocation of nuclear factor kappa B and release of inflammatory cytokines were also observed. These changes were accompanied by GSH-dependent redox imbalance and alterations in mitochondrial membrane potential and respiratory complexes enzyme activities leading to mitochondrial respiratory dysfunction, reduced ATP synthesis, and intrinsic caspase-9 mediated apoptosis. Caspase-9 was activated due to alterations in Bcl-2 and Bax proteins and release of cytochrome c into the cytosol. The activities of oxidative stress-sensitive mitochondrial matrix enzymes, aconitase, and glutamate dehydrogenase were also inhibited. Treatment with AZD showed beneficial effects on the recovery of antioxidant enzymes, inflammatory responses, and mitochondrial functions. GSH-dependent redox homeostasis also recovered after the treatment with AZD. This study may help in better understanding the etiology and pathogenesis of inflammation-induced disorders in pancreatic beta cells to better manage therapeutic strategies.

## Introduction

Oxidative stress and inflammation have been closely associated with numerous metabolic disorders. Inflammation-induced oxidative stress and cellular responses in the pancreas cause β-cell dysfunction, mass reduction (e.g., apoptosis) with consequential insufficiency in insulin secretion and/or action (Matsuda and Hattori, 2006; Ghonime et al., 2014; Li et al., 2018). Autophagy/mitophagy is important for the maintenance and protection of the function and morphology of pancreatic cells (Chen et al., 2014; Fan et al., 2018; Zhu et al., 2019). Immunological responses, metabolic adaptation, and mitochondrial dysfunction, in particular, are the hallmarks of inflammation-induced changes. Metabolic inflammation induced during lipotoxicity as seen in diabetes and obesity has also been implicated in the development of β-cell toxicity and metabolic complications (Chen et al., 2019). High glucose and fatty acid levels (glucolipotoxicity) in diabetes as well as exposure to pro-inflammatory bacterial endotoxin, lipopolysaccharide (LPS), could cause destruction of the pancreatic cells and alterations in mitochondrial function (Alnahdi et al., 2019a, 2020; Xu et al., 2022). Studies have shown that LPS acts synergistically with palmitic acid in altering β-cell function by activating the signaling of Toll-like receptor 4/nuclear factor-κB (TLR4/NF-κB), increasing reactive oxygen species (ROS) production and metabolic dysfunction (Pan et al., 2018; Xu et al., 2022). Mitochondrial autophagy (mitophagy) is a critical defense mechanism for the selective removal of dysfunctional or redundant mitochondria, thereby restricting the secretion and responses of inflammatory cytokines (Xu et al., 2020). LPS-induced mitochondrial damage, mitochondrial-dependent apoptosis, and mitophagy have been demonstrated to be protected by anti-oxidative and anti-inflammatory drugs by removing impaired mitochondria having low mitochondrial membrane potential and causing high ROS production (Wang et al., 2019). Under inflammatory conditions, such as a high-fat diet, the endogenous LPS produced by the gut microbiota increases the level of LPS in circulation and triggers metabolic changes associated with inflammatory responses and insulin resistance (Reilly et al., 2021).

Azadirachtin (AZD), a potent bioactive compound extracted from the medicinal Neem tree (*Azadirachta indica*) has long been known for its use as a pesticide, and also as a potent anti-inflammatory and anti-cancer agent (Fernandes et al., 2019). AZD has been reported to target multiple pathways that are aberrant in inflammation, diabetes, and cancer by modulating multiple cell-signaling pathways involved in cellular growth and apoptosis (Hao et al., 2014; Nagini, 2014; Dubey et al., 2019). It has been reported that AZD causes mitochondrial-dependent apoptosis and autophagy *in vitro* and hence facilitates the clearance of dysfunctional or damaged mitochondria, thus enhancing the protection against inflammation and oxidative stress-induced toxicities (Priyadarsini et al., 2010; Srivastava et al., 2012).

Increased oxidative stress and reduced antioxidant defense potential in the pancreas lead to β-cell apoptosis and defects in insulin secretion. Our recent study on LPS-induced insulin-secreting pancreatic Rin-5F cells showed increased oxidative stress and apoptosis. AZD treatment attenuated the ROS-dependent DNA damage, oxidative stress, and apoptosis, and enhanced the autophagy signaling (John and Raza, 2021). Our present study further investigated the protection of LPS-induced redox stress, inflammatory responses, and mitochondrial dysfunction in Rin-5F cells by AZD. Our results show that AZD treatment suppressed NF-κB signaling, which in turn, attenuated the inflammatory responses caused by LPS and protected the β-cells from caspase/Bcl-2-dependent apoptosis by restoring the glutathione-dependent redox homeostasis and mitochondrial bioenergetic function.

## Materials and Methods

Azadirachtin (AZD), LPS, reduced and oxidized glutathione (GSH/GSSG), 1-chloro 2,4-dinitrobenzene (CDNB), cumene hydroperoxide, glutathione reductase, NADH, NADPH, cytochrome c, coenzyme Q2, antimycin A, dodecyl maltoside, and ATP assay kits were procured from Sigma (St Louis, MO, United States). Kits for nitric oxide (NO) assays and mitochondrial membrane potential were procured from R & D Systems (MN, United States) and for lipid peroxidation (LPO) and aconitase from Oxis Int, Inc. (Portland, OR, United States). GSH/GSSG assay kits were purchased from Promega Corp. (Madison, WI, United States). Tumor necrosis factor-alpha (TNF-α) and interleukin-6 (IL-6) were procured from BD Pharmingen (BD Biosciences, San Jose, United States) while the phospho-NF-κB p65 sandwich ELISA kits were purchased from Cell Signaling Technology, Inc (Danvers, MA, United States). Kits for superoxide dismutase (SOD), glutamate dehydrogenase (GDH), and caspase-9 activity assays were purchased from Abcam (Cambridge, England, United Kingdom) while those for catalase and cyclooxygenase-2 (Cox-2) activity were procured from Cayman (MI, United States). Rin-5F cells were obtained from the American Type Culture Collection (Manassas, VA, United States). Polyclonal antibodies against NF–κB p65, I–κB, Bax, and VDAC were purchased from Cell Signaling Technology, Inc. (Danvers, MA, United States). Monoclonal antibodies against cytochrome c, cytochrome c oxidase (COX), Bcl–2, and actin were purchased from Santa Cruz Biotechnology Inc (Santa Cruz, CA, United States) while those against aconitase, cyclooxygenase-2 (Cox-2), and histone H3 were purchased from Abcam (Cambridge, England, United Kingdom). Reagents for cell culture, SDS-PAGE, and Western blot analyses were procured from Gibco BRL (Grand Island, NY, United States) and Bio-Rad Laboratories (Richmond, CA, United States).

### Propagation and Experimental Treatment of Cells

Rin-5F cells, a clone derived from the RIN-m rat pancreatic islet cell line, which produces and secrete insulin, were cultured in RPMI-1640 with 10% fetal bovine serum in a 37°C humidified incubator in the presence of 5% CO_2_—95% air. Once confluent (about 80%), cells were treated with bacterial endotoxin, LPS (1 μg/ml) for 24 h, and appropriate controls were used as described before (Nahdi et al., 2017; Al-Nahdi et al., 2018; Alnahdi et al., 2020). In addition, some of the cells were incubated with azadirachtin (AZD) (25 µM for 24 h), either alone or with LPS. Concentrations and time points for LPS and AZD used in the present study were based on literature search as well as our previous studies (Raza et al., 2014, 2016; John and Raza, 2021). After treatment, cells were harvested and homogenized in cold H-medium buffer (70 mM sucrose, 220 mM mannitol, 2.5 mM HEPES, 2 mM EDTA, and 0.1 mM phenylmethylsulfonyl fluoride, pH7.4). Sub-cellular fractionation was performed to isolate the nuclear, mitochondrial, and post-mitochondrial fractions as described previously (Nahdi et al., 2017; Al-Nahdi et al., 2018; Alnahdi et al., 2020), and protein concentration of the individual fractions was checked by the Bradford method (Bradford, 1976).

### Measurement of Apoptosis Markers

In our previous study, we observed LPS-induced caspase-mediated apoptosis in Rin-5F cells via the caspase-8 extrinsic pathway (John and Raza, 2021). To further elucidate the involvement of the intrinsic pathway, Rin-5F cells were treated with LPS and/or AZD and caspase-9 activity was measured as per the manufacturer’s protocol. Briefly, cell lysates from the control and treated cells were incubated with the caspase-specific substrate, LEHD conjugated to a chromophore, p-nitro aniline. The release of the chromophore was then measured spectrophotometrically at 405 nm as described in the manufacturer’s protocol (Abcam, Cambridge, England, United Kingdom).

Further confirmation of apoptosis was ascertained in the cells treated with LPS and/or AZD by evaluating the expression of apoptotic marker proteins, Bcl-2 and Bax, as well as the release of mitochondrial cytochrome c by SDS-PAGE and Western blot analysis (as described later).

### Measurement of Oxidative Stress

Membrane lipid peroxidation was measured to assess the oxidative stress in LPS and/or AZD-treated Rin-5F cells using the LPO-586 kit and the concentration of malondialdehyde was calculated as described before (Alnahdi et al., 2019b).

Alterations in nitric oxide (NO) production in the control and treated cells were assessed based on NO synthase (NOS) enzyme activity, using the NO assay kit (R & D Systems, MN, United States) which measures the nitrite formation using Griess reagent.

Antioxidant enzymes in the control and LPS and/or AZD-treated cells were measured using appropriate kits according to the manufacturer’s protocols. The catalytic action of catalase was measured based on the oxidation of methanol by hydrogen peroxide, in the presence of a chromogen, Purpald. The formaldehyde formed was then measured colorimetrically at 540 nm.

The conversion of xanthine to uric acid and hydrogen peroxide by xanthine oxidase was used as the basis for the measurement of SOD. SOD inhibited the reduction of NBT (nitro blue tetrazolium) to NBT-diformazan by superoxide ions, which was linearly related to the xanthine oxidase activity. SOD activity was thus measured as the percentage inhibition of NBT-diformazan formation.

### GSH Metabolism and Redox Homeostasis Measurement

GSH-redox homeostasis and metabolism were measured in control and LPS and/or AZD-treated Rin-5F cells. The ratio of GSH/GSSG (reduced glutathione to oxidized glutathione) was measured using the GSH/GSSG-Glo kit as per the vendor’s instructions as described before (Alnahdi et al., 2019b). Glutathione peroxidase (GSH–Px), glutathione reductase (GSH-reductase), and glutathione S-transferase (GST) activities were measured using cumene hydroperoxide, GSSG/NADPH, and CDNB as respective substrates, using standard protocols (Alnahdi et al., 2019b).

### Measurement of Inflammatory Markers

Media from control and LPS and/or AZD-treated Rin-5F cells was used for the measurement of inflammatory markers, TNF-α, and IL-6 using standard ELISA kits from BD Pharmingen (BD Biosciences, San Jose, United States) as described in the manufacturer’s protocols.

Cell lysates from LPS and/or AZD-treated cells were added to phospho-NF-κB p65 antibody-coated wells and incubated with NF-κB p65 antibody to detect the phospho-NF-κB p65 protein using the PathScan^®^ Phospho-NF-κB p65 sandwich ELISA assay kit as per the manufacturer’s protocol. Absorbance was read at 450 nm.

The peroxidase activity of the Cox enzyme was used as a basis for the measurement of cyclooxygenase -2 (Cox-2) activity in the cell lysates from control and LPS and/or AZD-treated cells. The assay was carried out in the presence of isozyme-specific inhibitors to distinguish Cox-2 from Cox-1 activity. The appearance of TMPD (N, N, N′, N′-tetramethyl-p-phenylenediamine) was monitored at 590 nm and used as a measure of Cox-2 activity.

### Measurement of Mitochondrial Function

Mitochondrial membrane potential was measured using a fluorescent cationic dye, DePsipher ™ (R & D Systems, MN, United States)in control and LPS and/or AZD-treated Rin-5F cells, and quantitated by flow cytometry as described before (Alnahdi et al., 2019a). The dye which could not access the transmembrane space due to low membrane potential remained in its green fluorescent form and this was measured using a flow cytometer (Becton Dickinson FACSCanto II).

### Measurement of Energy Metabolism

Freshly isolated mitochondria (5 µg protein) from control and LPS and/or AZD-treated Rin-5F cells were suspended in mitochondrial buffer (20 mM potassium phosphate buffer, pH 7.4 containing 0.2% lauryl maltoside). Mitochondrial respiratory enzymes, NADH-ubiquinone oxidoreductase (NADH-dehydrogenase, Complex I), succinate-ubiquinone oxidoreductase/ubiquinol-cytochrome c oxidoreductase (Complex II/III), and cytochrome c oxidase (Complex IV) were measured using their respective substrates, coenzyme Q_2,_ succinate/cytochrome c, and reduced cytochrome c, according to the method of Birch-Machin and Turnbull (Birch-Machin and Turnbull, 2001).

The ATP content in the LPS and/or AZD-treated cells was assayed using an ATP Bioluminescent cell assay kit according to the vendor’s protocol. The luminescence emitted was then measured using the TD-20/20 Luminometer (Turner Designs, Sunnyvale, CA, United States).

The activities of aconitase, and glutamate dehydrogenase (Krebs’ cycle enzymes), were measured using the aconitase assay kit (Oxis Int, Inc. Portland, OR, United States) and the glutamate dehydrogenase kit (Abcam, Cambridge, England, United Kingdom) respectively as per the vendor’s protocol as described before (Alnahdi et al., 2019a; Alnahdi et al., 2020).

### SDS-PAGE and Western Blot Analysis

Protein (5–10 µg) from the total, mitochondrial, nuclear, and post-mitochondrial extracts from control and treated Rin-5F cells were separated electrophoretically and transferred onto nitrocellulose membrane by Western blotting as described before (Alnahdi et al., 2019a; Alnahdi et al., 2020). Ponceau S staining was used to confirm equal loading of the transferred proteins and then probed with cytochrome c, Bax, Bcl-2, NF-κB p65, I-κB, Cox-2, cytochrome c oxidase (COX), and aconitase antibodies. Immunoreactive proteins were detected by enhanced chemiluminescence using the Sapphire Biomolecular Imager (Azure biosystems, Dublin United States) or using X-ray films. Loading controls used for total/post-mitochondrial, mitochondrial and nuclear fractions were beta-actin, VDAC, and histone H3, respectively. Densitometric analysis was done using Image Studio Lite Ver.5.2 (LI-COR Biosciences, Lincoln, Nebraska, United States) software, and histograms were plotted based on the relative ratios of the proteins normalized to their respective loading control.

### Statistical Analysis

The histograms plotted are expressed as mean ± SD of three independent experiments. The statistical difference between the groups was assessed by two-way ANOVA followed by LSD (least significant difference) posthoc analysis for multiple comparisons using SPSS software (version 23). *p*-values < 0.05 were considered statistically significant.

## Results

### LPS-Induced Apoptosis and Attenuation by AZD in Rin-5F Cells

**FIGURE 1:**
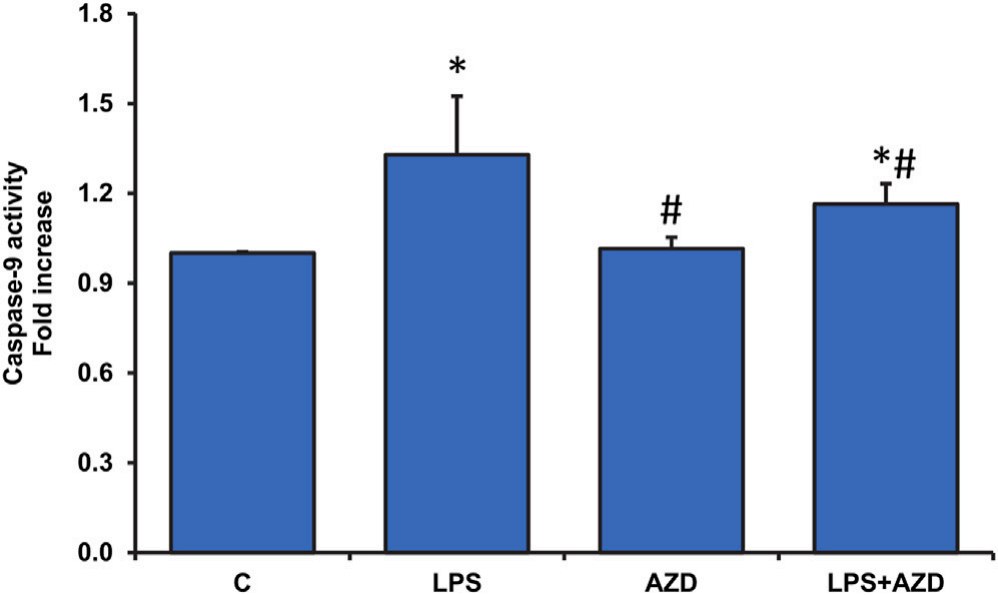
LPS-induced activation of caspase-9 activity and attenuation by AZD. Caspase-9 activity was measured in Rin-5F cells treated with LPS with/without AZD for 24 h, using the caspase-9 activity assay kit. Data are expressed as mean ± SD of three independent experiments. Statistical significances are shown as asterisks (*indicates significant difference (*p* ≤ 0.05) relative to control untreated cells whereas # indicates significant difference (*p* ≤ 0.05) relative to LPS-treated cells).

**FIGURE 2:**
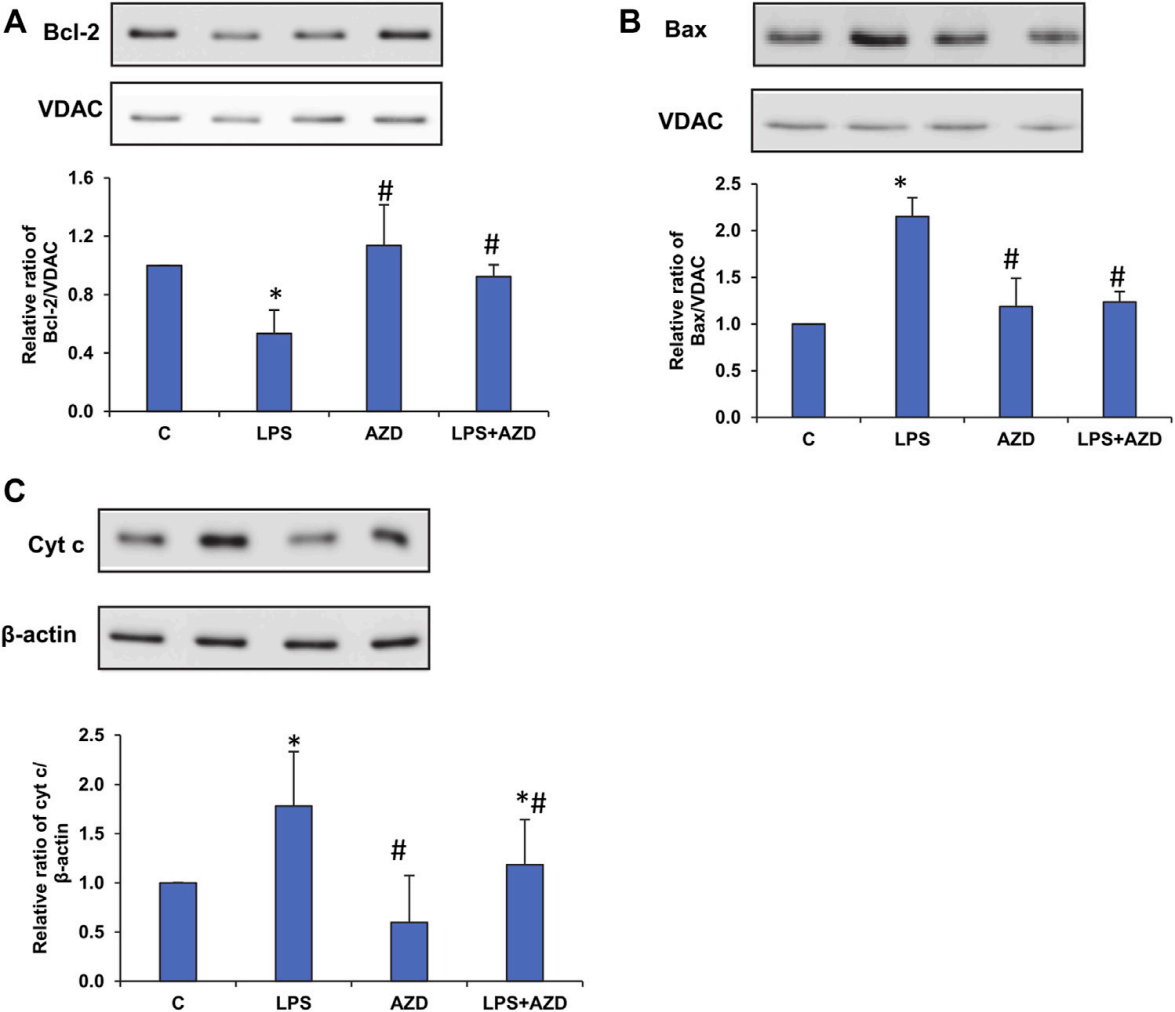
LPS-induced expression of mitochondrial oxidative stress markers. Mitochondrial and post-mitochondrial fractions (5–10 µg protein) from LPS and/or AZD-treated Rin-5F cells were separated electrophoretically and transferred onto nitrocellulose membranes by Western blotting. Immunoreactive proteins were probed using the respective antibodies against Bcl-2 **(A)**, Bax **(B)**, and post-mitochondrial (PMS) cytochrome c **(C)**. Bands were detected by enhanced chemiluminescence using the Sapphire Biomolecular Imager (Azure biosystems, Dublin, United States) or developed using X-ray films. VDAC and β-actin were used as loading controls for mitochondrial and post-mitochondrial fractions, respectively. Proteins were quantitated and normalized against their respective loading controls and represented as histograms. A representative of three independent experiments is given. Statistical significances are shown as asterisks (* indicates significant difference (*p* ≤ 0.05) relative to control untreated cells whereas # indicates significant difference (*p* ≤ 0.05) relative to LPS-treated cells).

Our recent study on Rin-5F cells (John and Raza, 2021) showed LPS-induced caspase-3/8-dependent apoptosis followed by increased DNA fragmentation in LPS treated cells and AZD caused attenuation of the apoptotic damages by enhancing the autophagic repair process. Our present study has further demonstrated that intrinsic caspase-9, which is the initiator caspase of the apoptotic pathway in the mitochondria, was also activated by LPS treatment and AZD protected the cells against LPS–induced apoptosis (Figure 1). This was further confirmed by analyzing the expression of Bcl-2 and Bax, oxidative stress marker proteins. A decrease in Bcl-2 expression was observed after LPS treatment which increased after AZD treatment (Figure 2A). Contrary to this, Bax expression was elevated in LPS-treated cells and AZD treatment brought the Bax protein level close to normal values (Figure 2B). Our results also indicated an increase in the release of mitochondrial intramembranous cytochrome c in the post-mitochondrial supernatant (PMS) (Figure 2C), indicating increased mitochondrial membrane permeability. These results are indicative of mitochondrial intrinsic apoptosis after LPS treatment and AZD appeared to be protective against LPS-induced apoptosis.

### Effects of AZD/LPS on Oxidative Stress and Redox Homeostasis in Rin-5F Cells

**FIGURE 3:**
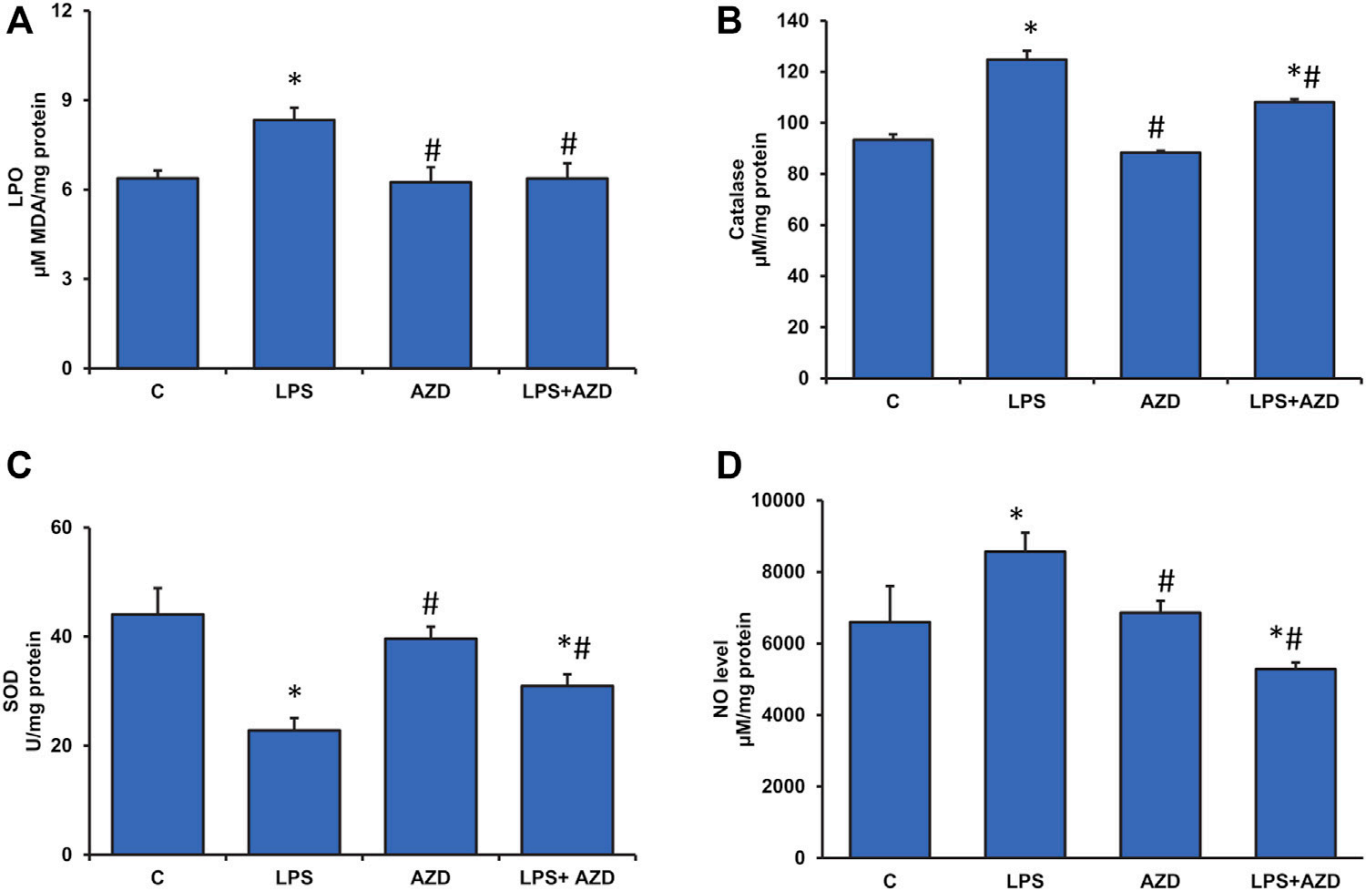
Assessment of oxidative stress in LPS-treated Rin-5F cells. Oxidative stress was assessed in LPS and/or AZD-treated Rin-5F cells using various oxidative stress parameters. LPO (lipid peroxidation) was measured as the total malondialdehyde produced **(A)** as per the manufacturer’s protocol. Catalase activity **(B)** was measured colorimetrically as the formaldehyde produced by oxidation of methanol by hydrogen peroxide. Percent inhibition of nitro blue tetrazolium (NBT)-diformazan was used as a measure of SOD activity **(C)** as per the vendor’s protocol. Total nitrite concentration was used as a measure of NO production **(D)** as per the manufacturer’s protocol. Data are expressed as mean ± SD of three independent experiments. Statistical significances are shown as asterisks (*indicates significant difference (*p* ≤ 0.05) relative to control untreated cells whereas # indicates significant difference (*p* ≤ 0.05) relative to LPS-treated cells).

In our previous study on Rin-5F cells (John and Raza, 2021), we also showed that LPS treatment caused a marked increase in ROS production, which leads to DNA damage and apoptosis. Our present study has further confirmed the consequences of high ROS production by measuring LPO and oxidative stress-sensitive enzymes, SOD, and catalase. Our results show that LPO was significantly increased (about 30%) after treatment with LPS and AZD brought the values close to the control level (Figure 3A). Cells treated with AZD only had minimal effect on LPO. Similarly, catalase activity was also elevated (almost 30%) in LPS-treated cells and the activity decreased significantly after AZD treatment, though still higher than control values (Figure 3B). Contrary to this, LPS treatment markedly decreased (almost 50%) the SOD activity, which increased significantly after AZD treatment, though slightly lower than control values (Figure 3C). Nitric oxide (NO), another marker for increased oxidative/nitrosative stress as well as inflammation was also found to be increased (about 30%) in LPS-treated cells and AZD protected the cells by decreasing the NO synthesis (Figure 3D).

**FIGURE 4:**
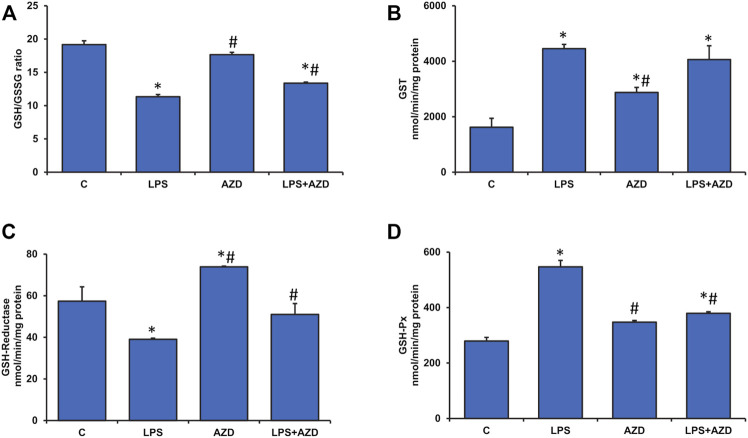
Alterations in redox homeostasis by LPS in Rin-5F cells. GSH/GSSG ratio **(A)**, GST **(B)**, GSH-reductase **(C)**, and GSH-Px **(D)** were measured in LPS and/or AZD-treated Rin-5F cells using the appropriate substrates. Data are expressed as mean ± SD of three independent experiments. Statistical significances are shown as asterisks (*indicates significant difference (*p* ≤ 0.05) relative to control untreated cells whereas # indicates significant difference (*p* ≤ 0.05) relative to LPS-treated cells).

Alterations in GSH-dependent redox homeostasis were also observed after LPS treatment. As shown in Figure 4A, the GSH/GSSG (reduced glutathione/oxidized glutathione) ratio was markedly reduced (almost 40%) after LPS treatment while AZD treatment showed slight recovery. AZD alone showed no change in the redox homeostasis. This was further evaluated by measuring the GSH-dependent metabolizing enzymes. Figure 4B shows almost a two-fold increase in GST activity after LPS treatment. The levels remained high even after AZD treatment. This could be due to the detoxifying activity of GST, since AZD alone also significantly increased GST activity, though not as much as LPS alone. We have also shown that LPS treatment inhibited the recycling of reduced GSH from oxidized GSSG by inhibiting the GSH-reductase enzyme activity (Figure 4C) and stimulating the GSH-Px activity (Figure 4D). Alterations in these GSH metabolizing enzyme activities, as well as redox homeostasis, appear to be protected by AZD treatment.

### LPS-Induced Inflammatory Response and Attenuation by AZD

**FIGURE 5:**
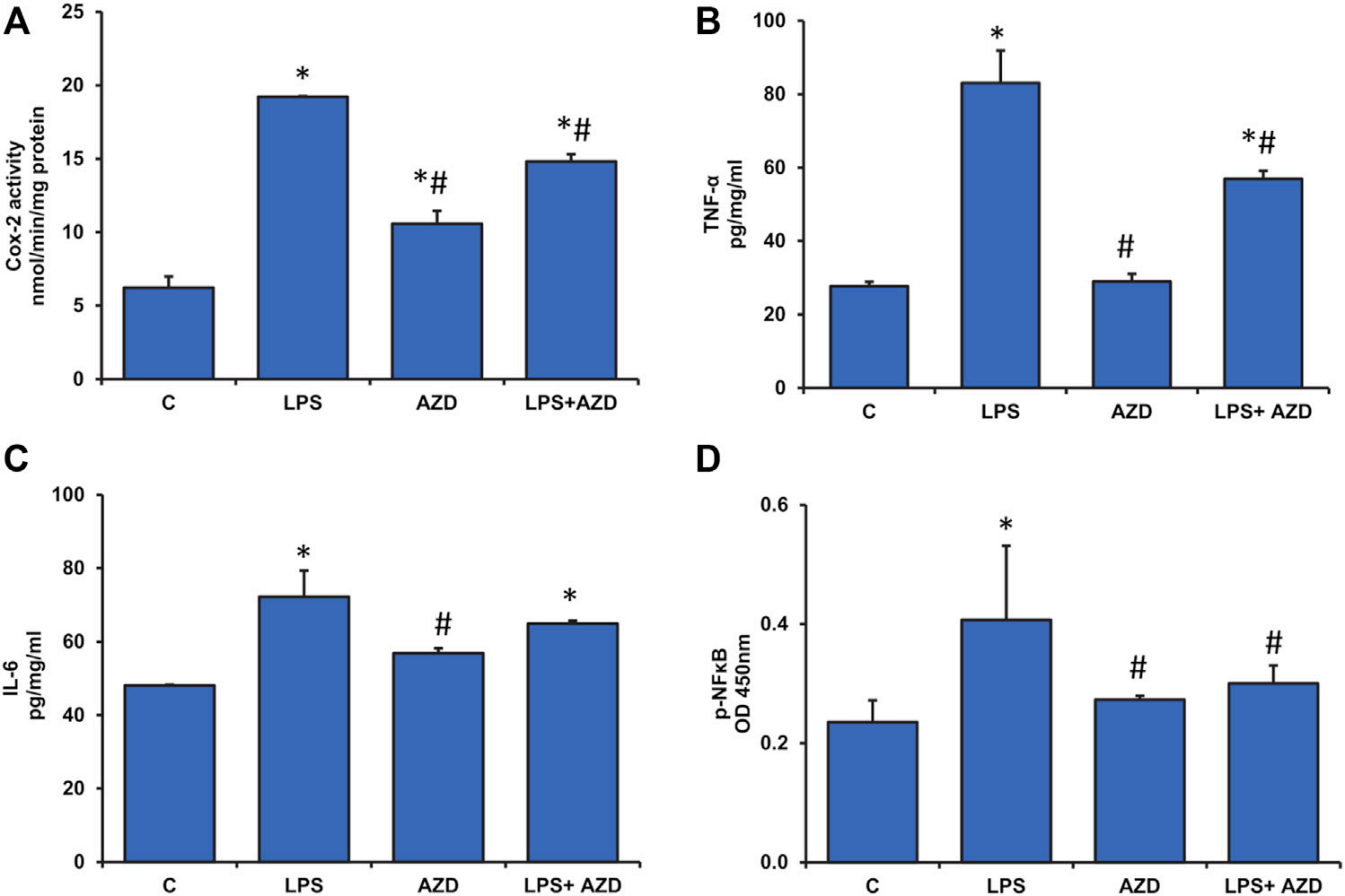
Production of inflammatory markers in Rin-5F cells after treatment with LPS. Cox-2 activity **(A)** was measured at 590 nm by examining the appearance of oxidized TMPD as per the vendor’s protocol. Other inflammatory markers like TNF-α **(B)**, IL-6 **(C)**, and p-NF-κB **(D)** were measured using standard ELISA kits as per the manufacturer’s protocol. Data are expressed as mean ± SD of three independent experiments. Statistical significances are shown as asterisks (*indicates significant difference (*p* ≤ 0.05) relative to control untreated cells whereas # indicates significant difference (*p* ≤ 0.05) relative to LPS-treated cells).

As seen earlier in Figure 3D, LPS treatment had increased NO production, which is a marker for increased oxidative/nitrosative stress as well as inflammation. To confirm this, we checked the levels of inflammatory markers, Cox-2, TNF-α, and IL-6. We observed a marked increase in Cox-2 activity after LPS treatment which was slightly inhibited by AZD treatment (Figure 5A). Increased levels of TNF-α (Figure 5B) and IL-6 (Figure 5C) were also observed in LPS-treated cells and AZD treatment significantly reduced the levels of these inflammatory cytokines. In support, we have also shown that LPS treatment increased NF-κB activation (as phosphorylated NF-κB) and this was protected by AZD treatment (Figure 5D). These results demonstrate that cells treated with LPS undergo immense inflammatory stress and AZD treatment partially protected the cells against LPS-induced inflammatory responses.

### Effects of LPS/AZD on Mitochondrial Functions

**FIGURE 6:**
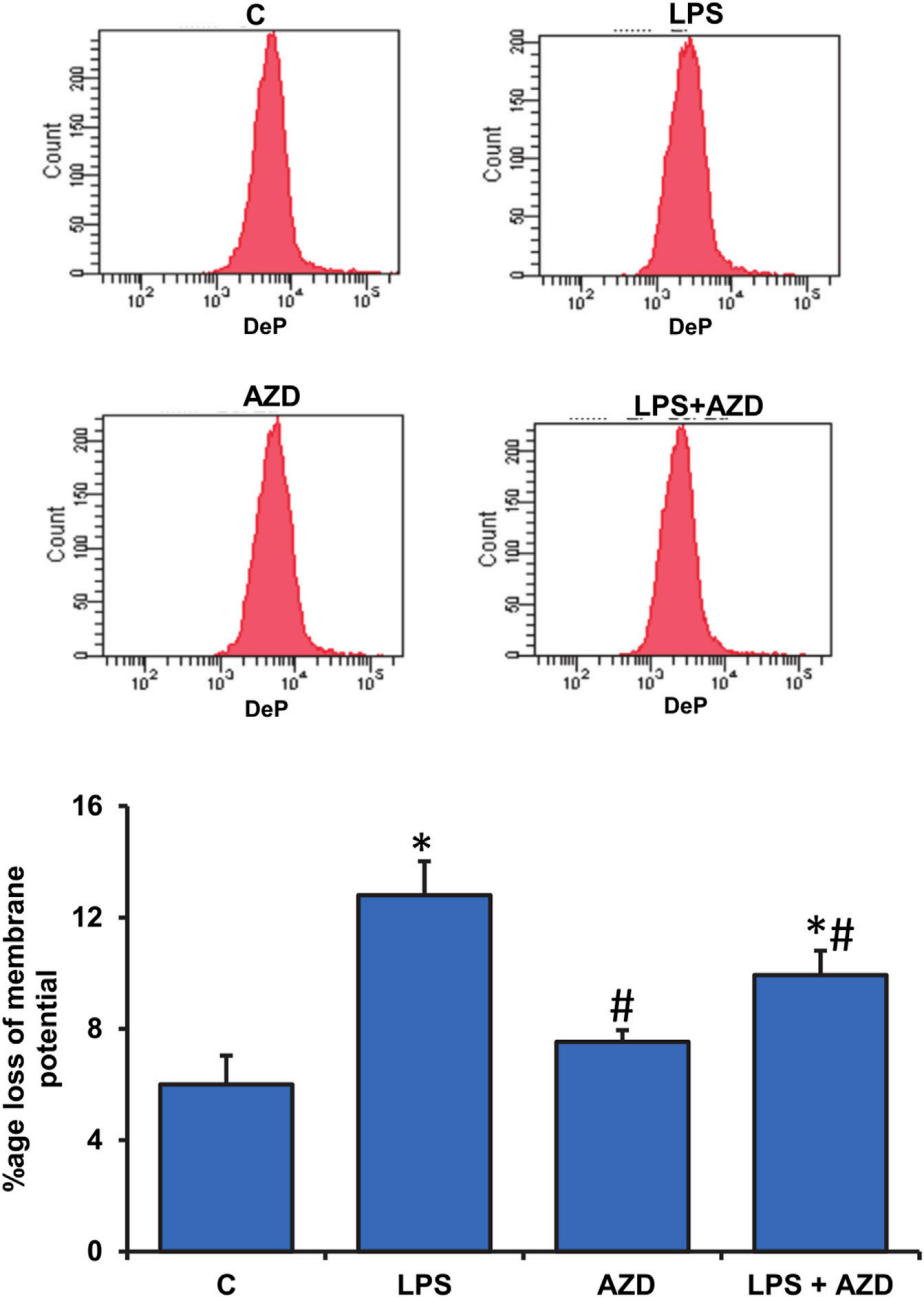
Alterations in mitochondrial membrane potential by LPS. Mitochondrial membrane potential was measured in Rin-5F cells treated with LPS and/or AZD by flow cytometry using a fluorescent dye, as per the vendor’s protocol. The histogram represents the percentage loss of mitochondrial membrane potential and represents the mean ± SD of three independent experiments. Statistical significances are shown as asterisks (*indicates significant difference (*p* ≤ 0.05) relative to control untreated cells whereas # indicates significant difference (*p* ≤ 0.05) relative to LPS-treated cells).

**FIGURE 7:**
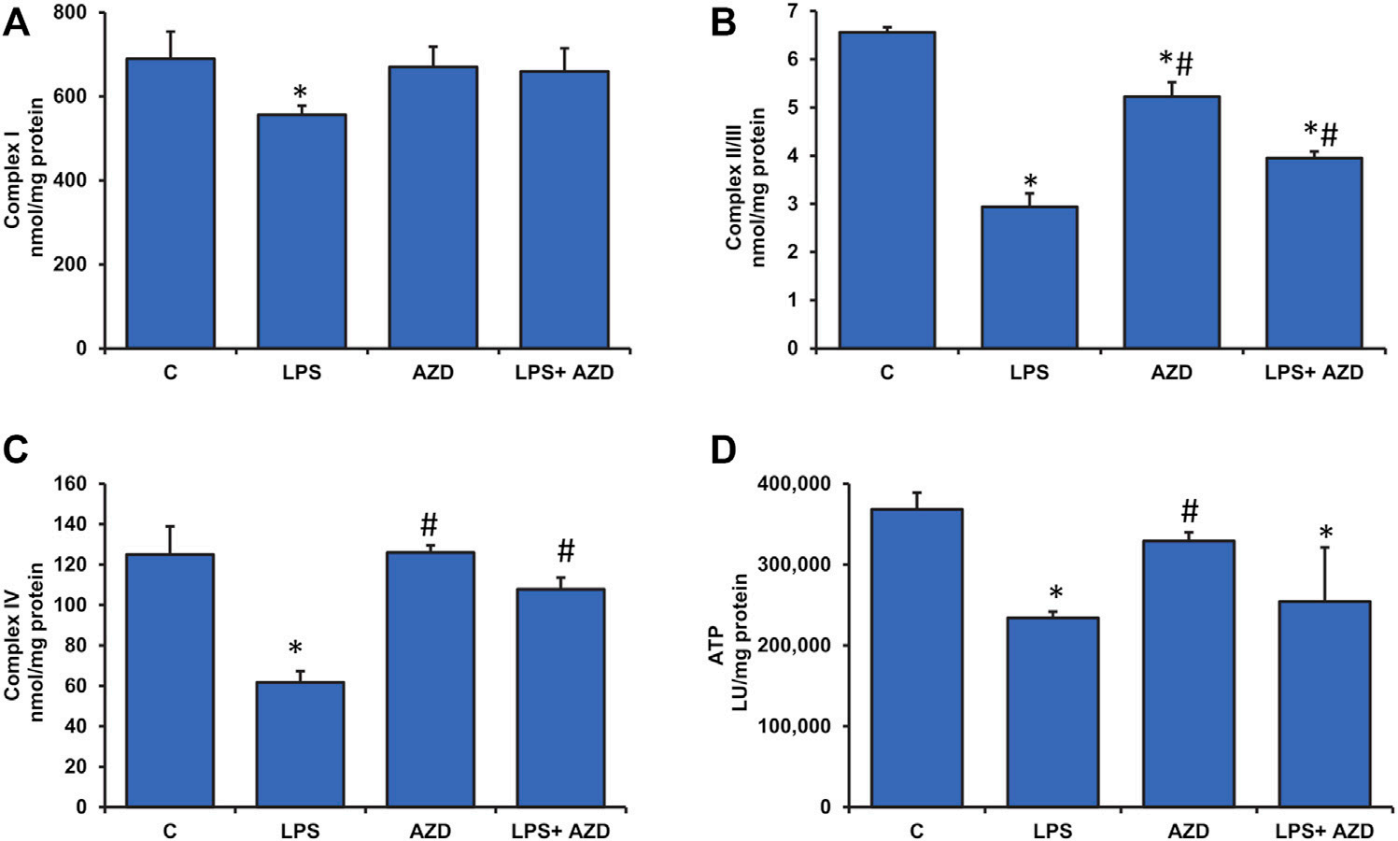
Alterations in the activities of mitochondrial respiratory complexes and bioenergetics caused by LPS in Rin-5F cells. Mitochondrial respiratory complexes, Complex I **(A)**, Complex II/III **(B)**, and Complex IV **(C)** were measured in Rin-5F cells treated with LPS and/or AZD, using their specific substrates. ATP levels **(D)** were measured using the ATP Bioluminescent cell assay kit as per the manufacturer’s protocol. Data are expressed as mean ± SD of three independent experiments. Statistical significances are shown as asterisks (*indicates significant difference (*p* ≤ 0.05) relative to control untreated cells whereas # indicates significant difference (*p* ≤ 0.05) relative to LPS-treated cells).

Significant disturbance in mitochondrial membrane potential was observed in LPS-treated Rin-5F cells, while AZD caused partial protection (Figure 6). LPS treatment also caused slight inhibition of mitochondrial complex I (around 20%) and a significant inhibition (around 50%) of complexes II/III and IV activities which were partially protected after AZD treatment (Figures 7A–C). In support, we also observed a significant reduction (almost 40%) in ATP production after LPS treatment (Figure 7D). However, only a moderate increase in ATP production was observed in cells treated with both LPS and AZD. A minimal decrease in ATP production was observed after AZD treatment alone.

**FIGURE 8:**
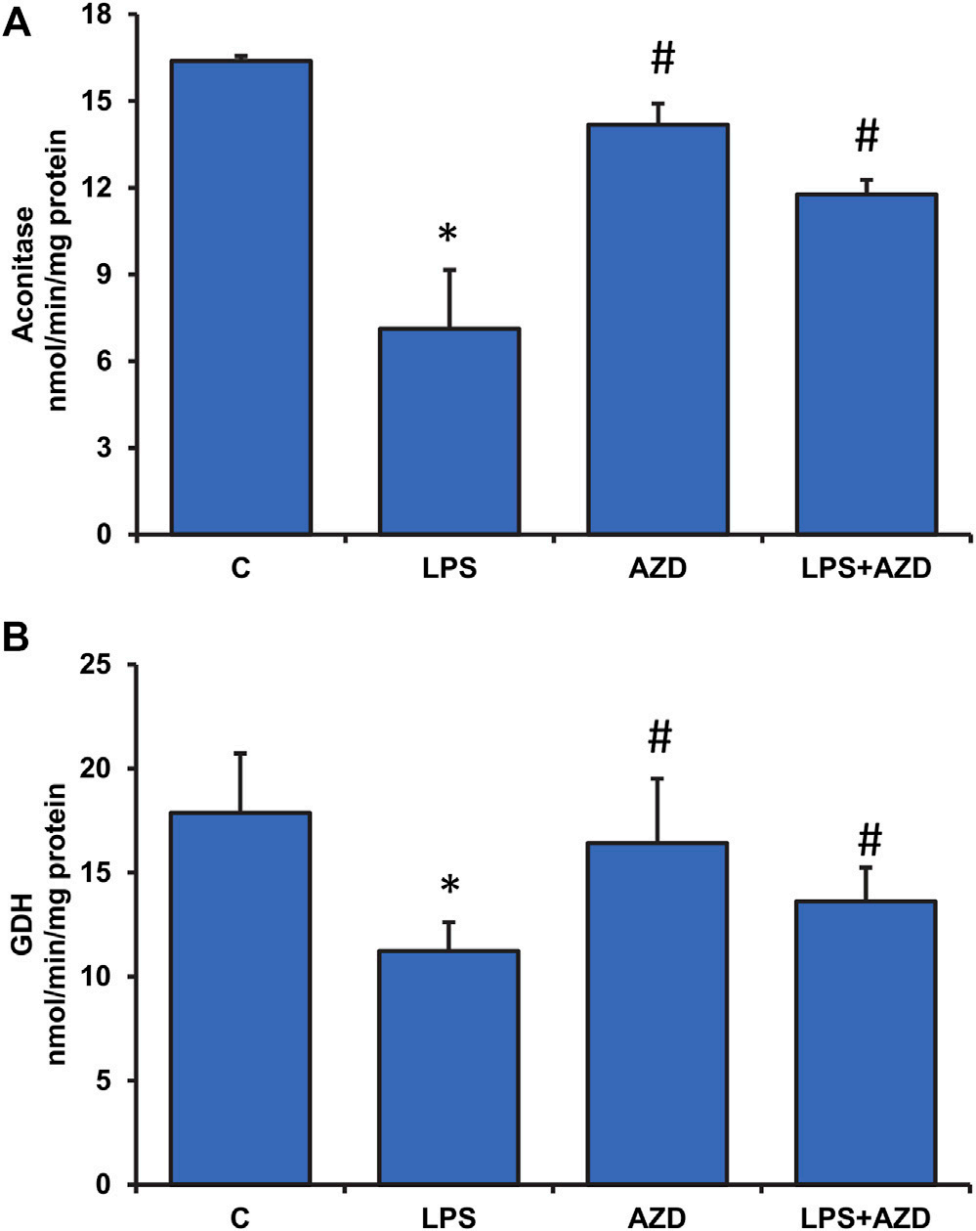
Effects of LPS on Krebs’ cycle enzymes in Rin-5F cells. Activities of Krebs’ cycle enzymes, aconitase **(A)**, and glutamate dehydrogenase **(B)**, were measured in LPS and/or AZD-treated Rin-5F cells using the aconitase assay kit (Oxis Int, Inc. Portland, OR, United States) and the glutamate dehydrogenase kit (Abcam, Cambridge, England, United Kingdom) respectively as per the vendors’ protocols. Data are expressed as mean ± SD of three independent experiments. Statistical significances are shown as asterisks (*indicates significant difference (*p* ≤ 0.05) relative to control untreated cells whereas # indicates significant difference (*p* ≤ 0.05) relative to compared with LPS-treated cells).

A significant inhibition (almost 60%) in the activity of ROS-sensitive aconitase, a mitochondrial matrix enzyme, was observed after LPS treatment in Rin-5F cells, which almost normalized after treatment with AZD (Figure 8A). AZD alone had minimal effect on the enzyme activity. Similarly, the activity of another mitochondrial matrix enzyme, glutamate dehydrogenase, which plays an important role in glutamate metabolism and energy homeostasis, was also markedly inhibited (about 40%) in LPS-treated cells, and AZD treatment brought the values close to control values (Figure 8B). Again, minimal effect was seen on AZD-treated cells.

### Expression of LPS-Induced Inflammatory and Apoptotic Markers and Protection by AZD

**FIGURE 9:**
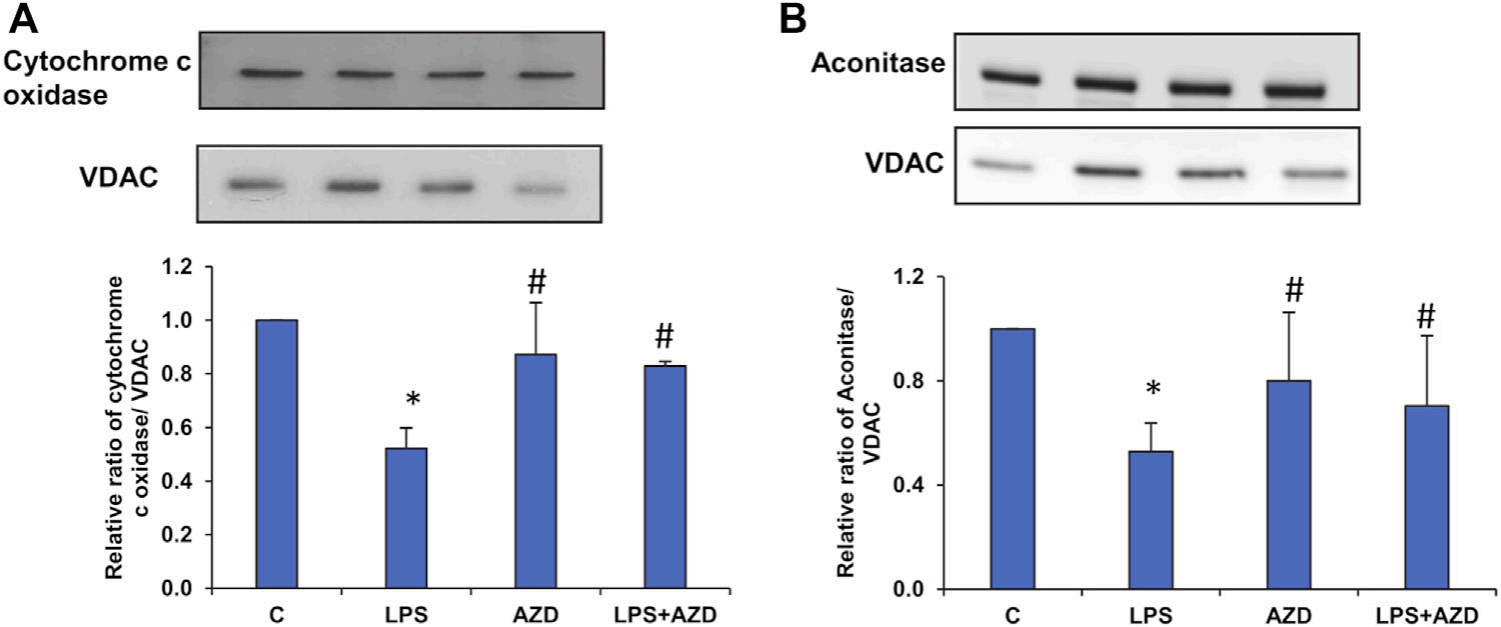
Expression of mitochondrial matrix markers after LPS treatment. Mitochondrial fractions (5–10 µg protein) isolated from Rin-5F cells treated with LPS and/or AZD were separated electrophoretically and transferred on to nitrocellulose membrane by Western blotting. Immunoreactive proteins were detected using the appropriate probes against cytochrome c oxidase **(A)**, and Aconitase **(B)**. Bands were detected by enhanced chemiluminescence using the Sapphire Biomolecular Imager (Azure biosystems, Dublin United States) or developed using X-ray films. VDAC was used as the loading control. Proteins were quantitated and normalized against their respective loading controls and represented as histograms. A representative of three independent experiments is shown. Statistical significances are shown as asterisks (* indicates significant difference (*p* ≤ 0.05) relative to control untreated cells whereas # indicates significant difference (*p* ≤ 0.05) relative to LPS-treated cells).

In support of our observation of the activities of mitochondrial enzymes, electrophoresis and Western blot analysis demonstrated a decrease in the expression of mitochondrial enzymes, cytochrome c oxidase (COX) (Figure 9A), and aconitase (Figure 9B) after treatment of Rin-5F cells with LPS, which were partially protected by AZD.

**FIGURE 10:**
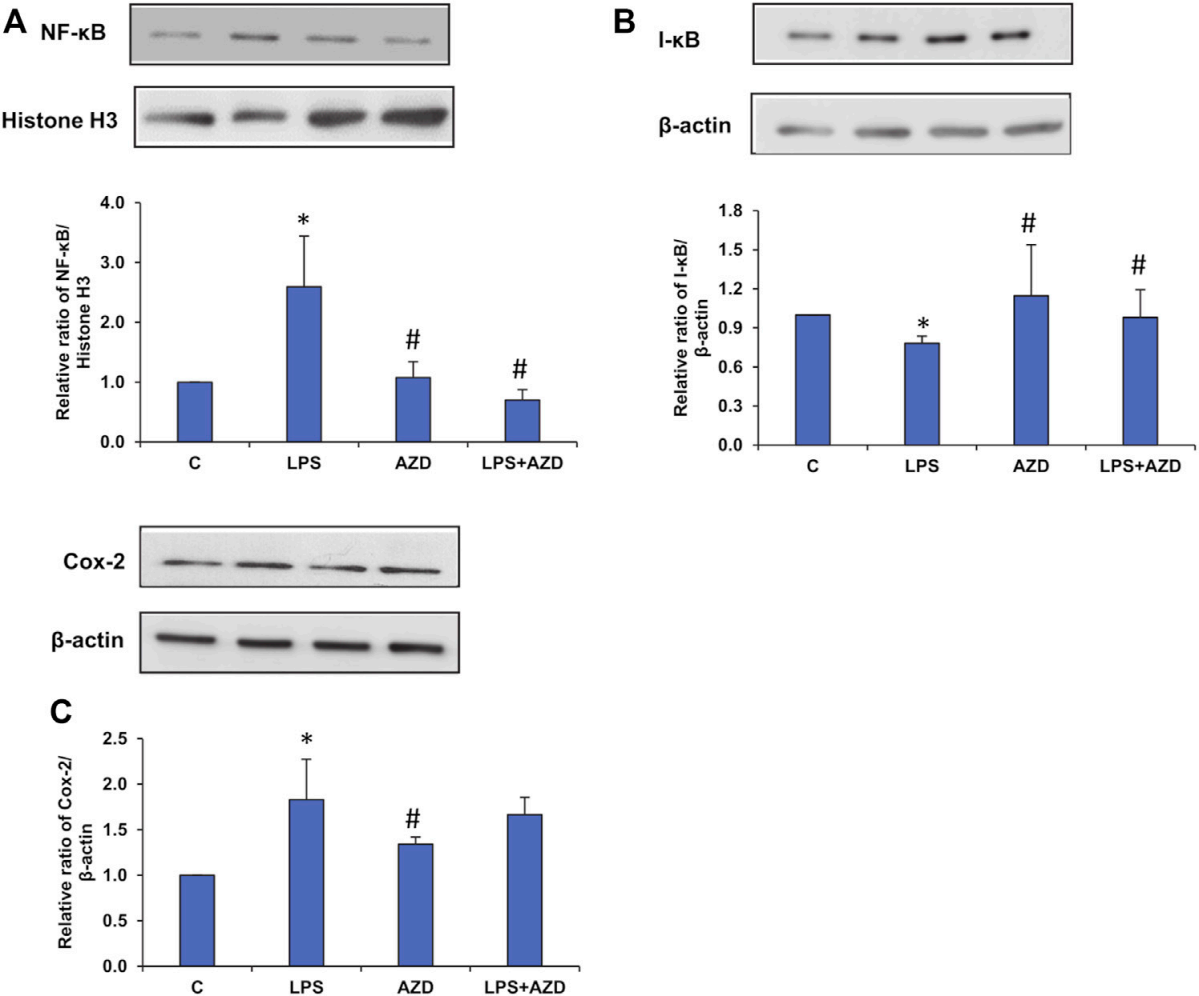
Expression of inflammatory markers induced by LPS. Nuclear, post-mitochondrial, or total cell extracts (25–30 µg protein) from LPS and/or AZD treated Rin-5F cells were separated electrophoretically and transferred onto nitrocellulose membranes by Western blotting. Immunoreactive bands were probed with the appropriate primary antibodies against NF-κB **(A)**, I-κB **(B)**, and Cox-2 **(C)**. Bands were detected by enhanced chemiluminescence using the Sapphire Biomolecular Imager (Azure biosystems, Dublin United States) or developed using X-ray films. Histone H3 and β-actin were used as loading controls for nuclear and post-mitochondrial/total extracts respectively. Proteins were quantitated and normalized against their respective loading controls and represented as histograms. A representative of three independent experiments is shown. Statistical significances are shown as asterisks (* indicates significant difference (*p* ≤ 0.05) relative to control untreated cells whereas # indicates significant difference (*p* ≤ 0.05) relative to LPS-treated cells).

Treatment with LPS induced translocation of NF-κB p65 into the nucleus (Figure 10A), which decreased after AZD treatment. Treatment with AZD significantly reduced the expression of the inflammatory marker. We also observed an increase in the cytosolic expression of inhibitory I-κB protein (Figure 10B), in LPS-treated cells confirming the release of the inhibitory protein from the NF-κB/I-κB complex and translocation of NF-κB to the nucleus. Increased expression of the inflammatory marker, cyclooxygenase-2 (Cox-2) (Figure 10C) was also observed after LPS treatment, which partially reduced in the presence of AZD.

## Discussion

Azadirachtin (AZD), a bioactive compound from the Neem tree, has been shown to have multiple beneficial functions in inflammation and oxidative stress-associated diseases such as cancer and diabetes (Dubey et al., 2019; Fernandes et al., 2019). Azadirachtin treatment on INS-1E (pancreatic β-cells) has been shown to protect the cells from oxidative stress-associated DNA damage and prevent mitochondrial and endoplasmic reticulum stress thus restoring glucose-stimulated insulin secretion (Dubey et al., 2019). This study also showed inhibition of amyloid (amylin) formation and disaggregation of pre-formed fibrils, which is a hallmark of islets in type 2 diabetic patients. Our recent study on Rin-5F cells demonstrated that AZD protected the cells from ROS-induced DNA damage and apoptosis induced by LPS treatment (John and Raza, 2021). Our study as well as other studies have suggested AZD to be a promising natural agent, that needs to be considered as a potential therapeutic compound in the management of diabetes, oxidative and inflammation-associated disorders. Our present study suggests that AZD repolarizes and reverses the pro-inflammatory responses triggered by LPS treatment thus preventing increased inflammatory signals in Rin-5F cells. The microbial products and cytokines released in inflammation and reversal of polarized inflammatory macrophages are potential targets in immunotherapy and anticancer treatment (Murray, 2017; Funes et al., 2018).

Exogenous (e.g., bacterial infections) or endogenous (e.g., high-fat diet and microbiome) increases in serum LPS have been shown to exhibit insulin resistance, impaired glucose tolerance, and severe dyslipidemia (Reilly et al., 2021). LPS production by the gut microbiome can accelerate the inflammatory pathways, inhibit the insulin signaling cascade and exacerbate the resulting metabolic defects (Peraldi and Spiegelman, 1998; Sangaran et al., 2021). Our present study which supports the findings, also demonstrates that LPS-treated Rin-5F β-cells are under severe oxidative and inflammatory stress, causing alterations in GSH-dependent redox homeostasis and alterations in mitochondrial bioenergetics. These changes have been partially attenuated by AZD treatment. We had earlier reported that AZD inhibits LPS-induced apoptosis, ROS production, DNA damage, and cell signaling pathways associated with apoptotic cell death (John and Raza, 2021). In our present study, we have further elucidated that LPS induced oxidative stress and inflammatory responses, as well as altered mitochondrial bioenergetics and redox homeostasis in Rin-5F cells, and were protected by AZD treatment. Studies have suggested the strong inhibitory action of AZD on pro-inflammatory cell signaling, cytokines, and NO activation via inhibition of nuclear translocation of NF-κB (Schumacher et al., 2011; Amyot et al., 2012; Lee et al., 2017). Our present study also demonstrates strong anti-inflammatory action by AZD via inhibition of oxidative stress, cytokine release as well as NF-κB-dependent cell signaling controlling the GSH-redox homeostasis. Induction of Cox-2, as well as NO synthesis by LPS, was also inhibited by AZD. Anti-oxidant and anti-inflammatory properties of Neem extract and AZD have also been well studied which was confirmed in our study (*Hao et al., 2014*; Islas et al., 2020). Studies have shown alterations in glutathione homeostasis and enhancement of antioxidant activity and anti-apoptotic mechanism after glutathione supplementation in LPS-induced animals (Zhu et al., 2007; Liu et al., 2020). Cells are known to control ROS levels by its elimination with the help of ROS-scavenging systems such as GSH-Px, GSH-reductase, SOD, and catalase (Jin et al., 2015). This was confirmed in our present study where LPS induced increased oxidative stress (LPO) and alterations in GSH/GSSG ratio and GSH-metabolizing enzymes, GSH-reductase and GSH-Px, which was brought to near normal by AZD treatment suggesting enhanced GSH recycling. Increased GST activity in LPS-treated cells may suggest an adaptation in the cellular ability to enhance detoxification of the toxin by utilizing GSH under oxidative stress conditions, which caused a reduction in the GSH levels in these cells. AZD treatment helped in restoring redox homeostasis. AZD also restored the alterations in the LPS-induced SOD and catalase activities.

LPS plays a crucial role in the constellation of sepsis, resulting in apoptotic damage to organelles, including mitochondria, that can modulate the intrinsic pathway via mediators activating caspase-9 (Haryanto et al., 2019). The activation of caspase-9, the initiator caspase of the mitochondrial apoptotic pathway, has been shown to be consistent with the appearance of mitochondrial dysfunction (Park et al., 2013; Molnár et al., 2021). Our study also demonstrated caspase-9 activation and mitochondrial dysfunction after LPS treatment, which was partially attenuated after AZD treatment. The Bcl-2 family of proteins, which include Bax and Bcl-2 proteins, which are pro-apoptotic and anti-apoptotic factors respectively, mainly regulate the intrinsic apoptotic pathway (Chang et al., 2013). Following a stress stimulus, Bax and Bcl-2 regulate cytochrome c release from the mitochondria, which in turn, activates caspase-9 (Chang et al., 2013). Our study also demonstrated an LPS-induced up-regulation of Bax and a downregulation of Bcl-2 accompanied by the release of cytochrome c from the mitochondria, resulting in activation of caspase-9. These effects were attenuated in the presence of AZD.

Metabolic stress due to increased mitochondrial dysfunction under oxidative and inflammatory conditions has been increasingly appreciated in diseases and toxicities (Bennett et al., 2021). Studies have revealed mitochondrial fragmentation and mitochondrial dysfunction caused by LPS-induced inflammatory responses *in vitro* as well as *in vivo* (Ma et al., 2018; Harland et al., 2020; Dong et al., 2021). In this study, we have demonstrated that LPS caused alterations in mitochondrial bioenergetics and respiratory functions in Rin-5F cells resulting in low ATP production. AZD treatment helped in partially restoring the mitochondrial respiratory functions, though the ATP level was still significantly lower than the control level. Mitochondrial oxidative stress was also observed after LPS treatment as the activity of aconitase, an iron-sulfur cluster enzyme sensitive to oxidative stress was markedly inhibited in Rin-5F cells after LPS treatment, and this effect was reversed by AZD treatment. Similarly, the activity of glutamate dehydrogenase (GDH), which plays an important role in glutamate metabolism and energy homeostasis, was also markedly inhibited in LPS-treated cells, and AZD treatment increased the activity. The mitochondrial enzyme GDH, which is an important link between anabolic and catabolic pathways, is also known to stabilize redox homeostasis in cells by activating the ROS scavenging enzyme, GSH-Px (Jin et al., 2015). GDH activation has also been shown to stimulate the citric acid cycle, resulting in activation of the respiratory enzyme complexes, causing increased ATP production (Pandya et al., 2019). Increased ATP production, in turn, is known to trigger insulin release in pancreatic β-cells (Göhring and Mulder, 2012).

**FIGURE 11:**
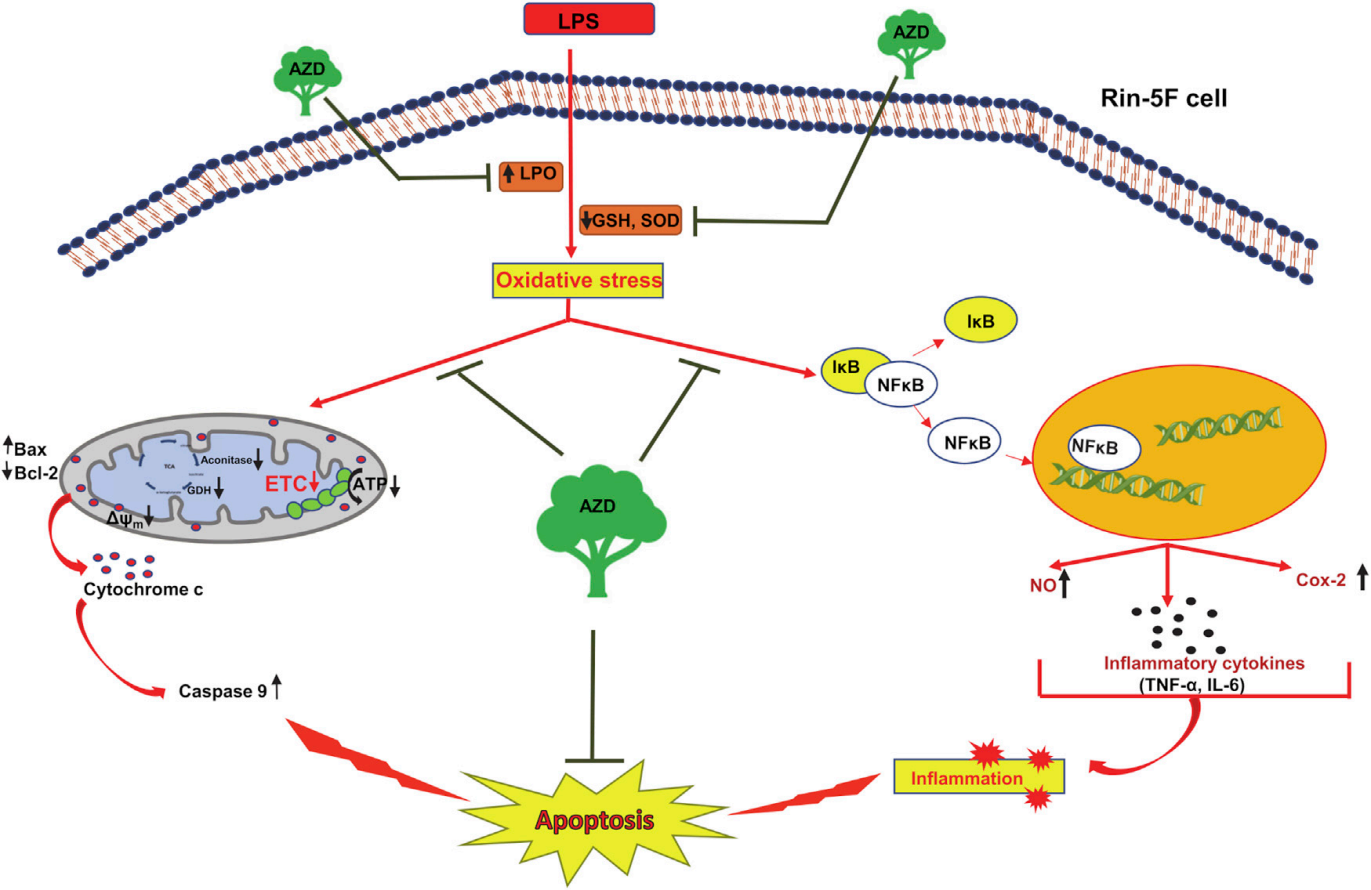
Schematic representation showing the protective mechanism of azadirachtin (AZD) on LPS-induced oxidative stress and inflammatory response in Rin-5F pancreatic cells. The bacterial endotoxin, lipopolysaccharide (LPS) has been shown to increase lipid peroxidation and decrease the physiological antioxidants, GSH (reduced glutathione) and SOD (superoxide dismutase), causing increased oxidative stress in the Rin-5F pancreatic cells. This, in turn, resulted in translocation of NF-κB into the nucleus, thus activating the inflammatory responses. LPS also caused decrease in the activities of mitochondrial respiratory complexes in the electron transport chain (ETC), causing alterations in the mitochondrial bioenergetics resulting in mitochondrial dysfunction, release of cytochrome c and caspase 9 activation leading to apoptosis. As illustrated in the model, AZD protected the cells from oxidative and mitochondrial stress by increasing the levels of GSH and SOD, increasing the activities of mitochondrial complexes and decreasing the inflammatory response, thus suppressing apoptosis.

In summary, we have further confirmed and elucidated that Rin-5F cells treated with LPS undergo severe oxidative and inflammatory stress accompanied by apoptotic changes and mitochondrial dysfunction. Our present study revealed that AZD treatment can protect LPS-induced cells from inflammation-related metabolic and molecular changes and mitochondrial dysfunction, thus enhancing cellular survival under extreme conditions of inflammatory and oxidative stress (Figure 11). Our findings might have implications in developing potential therapeutic strategies for inflammation and oxidative stress-associated degenerative processes, particularly in pancreatic β-cells, which may have relevance to diabetes-associated complications.

## Data Availability

The original contributions presented in the study are included in the article/Supplementary Material, further inquiries can be directed to the corresponding author.
